# Variability between human experts and artificial intelligence in identification of anatomical structures by ultrasound in regional anaesthesia: a framework for evaluation of assistive artificial intelligence

**DOI:** 10.1016/j.bja.2023.09.023

**Published:** 2023-10-27

**Authors:** James S. Bowness, Robert Morse, Owen Lewis, James Lloyd, David Burckett-St Laurent, Boyne Bellew, Alan J.R. Macfarlane, Amit Pawa, Alasdair Taylor, J. Alison Noble, Helen Higham

**Affiliations:** 1 Nuffield Department of Clinical Anaesthesia, University of Oxford, Oxford, UK; 2 Department of Anaesthesia, Aneurin Bevan University Health Board, Newport, UK; 3 Intelligent Ultrasound, Cardiff, UK; 4 Department of Anaesthesia, Royal Cornwall Hospitals NHS Trust, Truro, UK; 5 Department of Surgery & Cancer, Imperial College London, London, UK; 6 Department of Anaesthesia, Imperial College Healthcare NHS Trust, London, UK; 7 Department of Anaesthesia, NHS Greater Glasgow & Clyde, Glasgow, UK; 8 School of Medicine, Dentistry & Nursing, University of Glasgow, Glasgow, UK; 9 Department of Anaesthesia, Guy's & St Thomas' NHS Foundation Trust, London, UK; 10 Faculty of Life Sciences and Medicine, King's College London, London, UK; 11 Department of Anaesthesia, NHS Tayside, Dundee, UK; 12 Institute for Biomedical Engineering, University of Oxford, Oxford, UK; 13 Department of Anaesthesia, Oxford University Hospitals NHS Foundation Trust, Oxford, UK

**Keywords:** artificial intelligence, machine learning, medical devices, regional anaesthesia, sono-anatomy, ultrasonography, validation

## Abstract

**Background:**

*ScanNav*^*TM*^*Anatomy Peripheral Nerve Block* (ScanNav™) is an artificial intelligence (AI)-based device that produces a colour overlay on real-time B-mode ultrasound to highlight key anatomical structures for regional anaesthesia. This study compares consistency of identification of sono-anatomical structures between expert ultrasonographers and ScanNav™.

**Methods:**

Nineteen experts in ultrasound-guided regional anaesthesia (UGRA) annotated 100 structures in 30 ultrasound videos across six anatomical regions. These annotations were compared with each other to produce a quantitative assessment of the level of agreement amongst human experts. The AI colour overlay was then compared with all expert annotations. Differences in human–human and human–AI agreement are presented for each structure class (artery, muscle, nerve, fascia/serosal plane) and structure. Clinical context is provided through subjective assessment data from UGRA experts.

**Results:**

For human–human and human–AI annotations, agreement was highest for arteries (mean Dice score 0.88/0.86), then muscles (0.80/0.77), and lowest for nerves (0.48/0.41). Wide discrepancy exists in consistency for different structures, both with human–human and human–AI comparisons; highest for sartorius muscle (0.91/0.92) and lowest for the radial nerve (0.21/0.27).

**Conclusions:**

Human experts and the AI system both showed the same pattern of agreement in sono-anatomical structure identification. The clinical significance of the differences presented must be explored; however the perception that human expert opinion is uniform must be challenged. Elements of this assessment framework could be used for other devices to allow consistent evaluations that inform clinical training and practice. Anaesthetists should be actively engaged in the development and adoption of new AI technology.

**Editor’s key points**

• Methods to evaluate and artificial intelligence (AI)-based adjuncts to ultrasound-guided regional anaesthesia have not been established.
• This study compares identification of sono-anatomical structures between expert ultrasonographers and the AI-based ScanNav^TM^ technology.
• Using a library of ultrasound images across six anatomical regions, 19 experts in ultrasound-guided regional anaesthesia annotated 100 structures which were compared with each other and to the AI-generated colour overlay.
• Human–human and human–AI agreement was highest for arteries and lowest for nerves, with wide differences in consistency for different structures.
• Human experts and the AI system showed the same pattern of agreement in sono-anatomical structure identification; this approach provides a model for future assessment and comparison of AI-assisted ultrasonography.

Ultrasound image guidance for regional anaesthesia, first described in 1989,^1^ is now the predominant technique used to direct the targeted blockade of peripheral nerves.^2^ Image interpretation is critical in this practice, including the accurate identification of key sono-anatomical structures.^3^ Recent guidance aims to standardise anatomical structure identification for safe and effective performance of ultrasound-guided regional anaesthesia (UGRA).^4^^,^^5^

Assistive artificial intelligence (AI) technology could have a role in UGRA through supporting ultrasound image interpretation,^6^ particularly for non-experts, and systems have begun to emerge that aid in the identification of key structures.^7^, ^8^, ^9^, ^10^, ^11^, ^12^, ^13^ Evaluation of such devices typically involves comparison to identification by a limited number of human experts (up to three), comparing the agreement of an AI-generated structure overlay with that derived from the human experts (the ‘ground truth’).^14^, ^15^, ^16^, ^17^ However, human image interpretation is known to be variable,^18^^,^^19^ thus a small number of individuals might not adequately represent the diversity of expert opinion. Furthermore, these quantitative assessments of system accuracy are unfamiliar to clinicians and lack clinical context, as there is no clear threshold at which overlap between an AI prediction and human expert ground truth is known to be clinically acceptable. Other studies report qualitative analysis of accuracy, using expert assessment of AI structure identification of real-time or pre-recorded ultrasound images.^7^^,^^8^^,^^11^ Typically, studies do not undertake both analyses for the same structures and ultrasound images, thus not maximising the opportunity to evaluate the system. Furthermore, different systems are evaluated on different ultrasound scans when visualising different structures: this limits comparison between one system and another.

This study quantitatively and objectively evaluates the variability in sono-anatomical structure identification by human experts. We compare this with AI algorithms used by a system recently approved for clinical use in Europe and the USA (*ScanNav*^*TM*^ *Anatomy Peripheral Nerve Block*; ScanNav™, Intelligent Ultrasound, Cardiff, UK). The aim of the study is to evaluate and report any differences in sono-anatomical structure identification when comparing human–human image analysis with human–AI analysis. These data are presented alongside a subset of published qualitative data^8^ derived from the same underlying ultrasound videos and AI system to provide clinical context. This is intended to provide a case study demonstrating the need for a consistent evaluation framework for novel AI devices in this field.

## Methods

Ethical approval for this study was granted by the Oxford University Medical Sciences Inter-Divisional Research Ethics Committee (R75449/RE001).

### Ultrasound scans

Ultrasound scans were obtained from our previous study^8^ in which UGRA experts collected 720 ultrasound scans of 10-s duration from healthy adult subjects (without known pathology affecting the areas scanned), with 80 scans performed for each anatomical region, using SonoSite ultrasound machines (Fujifilm SonoSite, Bothell, WA, USA) with an X-Porte HFL50xp/L38xp linear or C60xp curvilinear probe, and PX L15-4 and L12-3 linear or C5-1 curvilinear probe. The scans were reviewed by three UGRA experts to ensure an appropriate ultrasound view was obtained and no atypical anatomy was present.

Scans from six of the anatomical regions in the study above, representing ‘basic’ (Plan A) UGRA procedures,^4^^,^^20^ were utilised: interscalene block (ISB) and axillary block (AxB) levels of the brachial plexus, erector spinae plane block (ESPB), rectus sheath block (RSB), adductor canal block (ACB), and popliteal level sciatic nerve block (SNB). Five scans for each anatomical region, displaying an appropriate view and without atypical anatomy, were sampled at random from the scans collected.

### Anatomical structures

Anatomical structures considered are strong recommendations for identification on ultrasound in the block view for each peripheral nerve block (see Supplementary material for table of structures by structure class and block region).^4^ Only the axillary vein was omitted, as this is not identified by the AI system in question. In total, 20 anatomical structures were considered across all anatomical regions. As five scans were included for each region, a total of 100 anatomical structures were evaluated across the 30 ultrasound scans.

### Expert reviewer assessment

Nineteen experts in UGRA (including five of the authors) were recruited from seven centres in the UK to assess the recorded ultrasound scans, providing a geographically diverse representation of practice. All were consultant anaesthetists practising in the UK National Health Service (NHS) and met at least two of the following criteria: completed advanced training in UGRA or held a UGRA-related qualification (e.g. European Diploma in Regional Anaesthesia & Acute Pain Management, higher degree); regularly delivered direct clinical care using UGRA (including for ‘awake’ surgery where indicated); and regularly taught UGRA (included advanced techniques).

Experts viewed the 30 ultrasound scans in the same predetermined random order on a HUION Kamvas Pro 13 Graphic Drawing Monitor (HUION, Shenzhen, China). At the end of each scan, the expert was able to view the final still frame image and use a stylus to annotate the required structures before moving on to the next scan (see Supplementary material for ‘instructions for annotation’). Thus, 19 experts annotated each of the 100 structures (but were blinded to the annotations of the other experts).

### Artificial intelligence device

A 20th assessment was derived from the AI-generated colour overlay produced by ScanNav™. ScanNav™ is an approved medical device in Europe and the USA that uses deep learning to produce a colour overlay on real-time ultrasound, highlighting anatomical structures of interest in UGRA (https://www.intelligentultrasound.com/scannav-anatomy-pnb/). The colour overlay for each structure on the final still frame image of the videos was used for comparison with the human expert annotations.

### Previous qualitative evaluation of artificial intelligence

In our previous study,^8^ three experts analysed the ScanNav™ colour overlay and provided a qualitative assessment of accuracy. Experts assessed whether the AI-generated colour overlay on each structure in that video was correct (true positive/negative, false positive/negative). The experts also assessed the potential for the AI overlay to modify the risk of adverse events (e.g. trauma to nerves, arteries, pleura, or peritoneum) and block failure. As each structure appeared in a maximum of five ultrasound videos, each assessed by three experts, a total of 15 individual assessments could be made. In addition, they provided a score (0=poor; 10=excellent) to rate the overall highlighting performance.^8^ Data from the relevant sono-anatomical structures in our previous study^8^ are presented here for comparison with the quantitative assessment data gathered in this study.

### Quantitative evaluation of human experts and artificial intelligence

**Fig 1:**
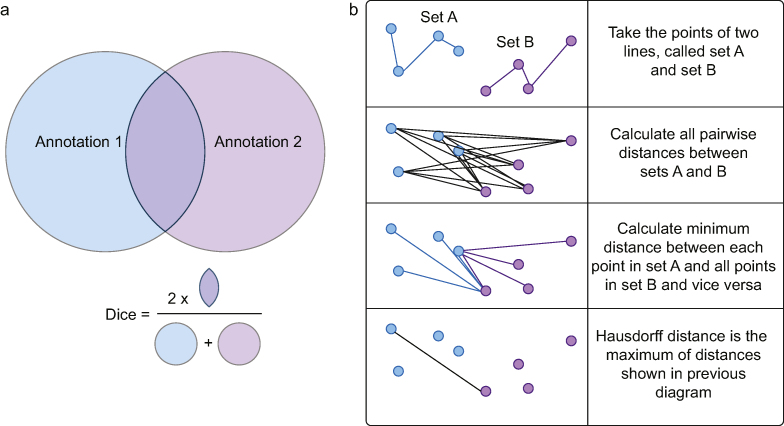
Illustration of Dice and Hausdorff metrics. (a) The Dice metric compares two enclosed areas. The area of overlap (multiplied by two) is divided by the total areas of the combined annotations. A higher figure indicates stronger agreement (0=no agreement, 1=complete agreement). (b) The Hausdorff metric compares two line annotations. The distance between each point in line A is compared to all points in line B. The minimum distance of this group is calculated. This is repeated for each point in line A. The maximum of these minimum values is then taken. A lower figure indicates stronger agreement (0=complete agreement, 1=lines separated by maximum length/width of image).

For each expert participant, any image without annotation for all structures was assumed to have been omitted in error and discarded from analysis. If a structure was not annotated, whereas at least one other structure in the image was, it was assumed to have been deemed ‘not visible’ on the image, and therefore included for analysis. Structure annotations that enclosed an area (arteries, muscles, and nerves) were compared using the Dice metric to allow comparison of the overlapping enclosed areas (Fig. 1a). Annotations that required a single line to denote a structure or tissue plane (fascial/serosal planes) were compared using the Hausdorff metric to allow evaluation of the degree of difference in the lines drawn (Fig. 1b).

Annotations for the C5 and C6 nerve roots were grouped together for analysis, as were the anterior and posterior layers of the rectus sheath, as this is the method by which the AI system was developed and so the predictions produced.

Human annotations for a given structure were initially compared with all other human annotations to provide an assessment of inter-observer variability for human experts. The mean, median, minimum–maximum range, and standard deviation (sd) of the Dice/Hausdorff metric for each class of structures are reported (artery, muscle, nerve, and fascia/serosal plane) and presented for each individual structure. The AI annotation for that structure was then compared with the human annotations, and reported in a similar manner to provide an assessment of AI–human variability. These were then compared with the qualitative analysis.

## Results

This study is reported according to the guidelines for early-stage clinical evaluation of decision support systems driven by AI (DECIDE-AI).^21^ Twenty subjects (10 male, 10 female) contributed to the 30 scans included. The average age of subjects was 41.75 yr (range 23–64) and BMI 28.3 kg m^−2^ (19.7–38.4; sd 5.5). Information for each subject and block is contained in the Supplementary material.

The 19 experts asked to annotate 100 structures provided a total of 1900 potential structure annotations. As the C5/C6 nerve roots and the anterior/posterior layers of the rectus sheath were each grouped together as a single structure for analysis, a maximum total of 1710 (19×90) structure annotations was possible. Annotations from one ISB image (three annotations; C5/6 nerve roots and two scalene muscles) and one ESPB image (two annotations; ESP muscle group and transverse process) were not recorded by one participant, and thus were removed from the analysis (total of five annotations omitted). Twenty-six structure annotations were omitted from analysis because of incorrect annotation (enclosure instead of line or *vice versa*). Thus, a total of 31 annotations were omitted or performed incorrectly by the experts, leaving a total of 1679 annotations collected and analysed.

Table 1 shows summary data for annotations by structure class (artery, muscle, nerve, and fascia/serosa) comparing human–human annotations and AI–human annotations. Mean Dice score was highest for artery annotations (muscle highest for median), followed by muscle, and then nerve, in both human–human and AI–human comparisons. Variation (sd) in structure identification was greatest for nerves, followed by muscles, and then arteries (also for both human–human and human–AI). Fascial/serosal planes cannot be directly compared with the other classes as they were the only class assessed using the Hausdorff metric. In each case, the mean score was improved (higher for Dice/lower for Hausdorff) for human–human comparisons than for human–AI comparisons, although human–AI comparisons showed less variation.

**Table 1:** Human–human (H-H) and human–artificial intelligence (H-AI) annotation comparisons by structure class.

Summary of comparisons					
Structure class	Min	Mean	Median	Max	sd
**Dice metric**					
Artery					
- H-H	0.00	0.88	0.89	0.97	0.10
- H-AI	0.00	0.86	0.89	0.96	0.09
Nerve					
- H-H	0.00	0.48	0.59	0.96	0.33
- H-AI	0.00	0.41	0.50	0.93	0.29
Muscle					
- H-H	0.00	0.80	0.90	0.98	0.23
- H-AI	0.00	0.77	0.84	0.97	0.23
**Hausdorff metric**					
Fascia/serosa					
- H-H	0.00	0.08	0.03	1.00	0.13
- H-AI	0.00	0.16	0.05	1.00	0.21

Table 2 shows data for annotations of each structure comparing human–human and AI–human annotations. Of the 18 structures considered (C5/C6 and anterior/posterior layers of rectus sheath combined), the mean score for human–human annotation was superior in 11, human–AI annotation was superior in six, and they were equal in one. However, human–AI comparisons had greater consistency (less variation/smaller sd) in 10 structures, compared with eight structures for human–human comparisons.

**Table 2:** Human–human (H-H) and human–artificial intelligence (H-AI) annotation comparisons by structure.

Comparisons by block and structure (Dice metric)					
Structure	Min	Mean	Median	Max	sd
**Interscalene block**					
C5 & C6					
- H-H	0.00	0.60	0.63	0.87	0.18
- H-AI	0.02	0.54	0.52	0.87	0.15
Anterior scalene					
- H-H	0.00	0.70	0.80	0.96	0.26
- H-AI	0.00	0.73	0.75	0.95	0.20
Middle scalene					
- H-H	0.00	0.67	0.75	0.95	0.25
- H-AI	0.00	0.70	0.75	0.94	0.19
**Axillary brachial plexus**					
Axillary artery					
- H-H	0.00	0.85	0.88	0.96	0.14
- H-AI	0.00	0.84	0.85	0.96	0.11
Median nerve					
- H-H	0.00	0.44	0.51	0.86	0.26
- H-AI	0.00	0.42	0.52	0.94	0.34
Musculocutaneous nerve					
- H-H	0.00	0.50	0.65	0.94	0.34
- H-AI	0.00	0.38	0.53	0.84	0.32
Radial nerve					
- H-H	0.00	0.21	0.00	0.94	0.30
- H-AI	0.00	0.27	0.24	0.74	0.25
Ulnar nerve					
- H-H	0.00	0.31	0.06	0.94	0.36
- H-AI	0.00	0.31	0.22	0.84	0.30
**Erector spinae plane block**					
ES muscle group					
- H-H	0.00	0.81	0.92	0.98	0.25
- H-AI	0.00	0.62	0.75	0.95	0.36
**Rectus sheath block**					
Rectus abdominis					
- H-H	0.75	0.92	0.93	0.98	0.04
- H-AI	0.65	0.87	0.91	0.97	0.08
**Adductor canal block**					
Femoral artery					
- H-H	0.74	0.90	0.90	0.97	0.04
- H-AI	0.73	0.88	0.89	0.96	0.05
Saphenous nerve					
- H-H	0.00	0.51	0.56	0.92	0.26
- H-AI	0.00	0.36	0.50	0.84	0.30
Sartorius					
- H-H	0.00	0.91	0.94	0.98	0.14
- H-AI	0.01	0.92	0.93	0.97	0.10
**Sciatic nerve block**					
Sciatic nerve					
- H-H	0.31	0.78	0.82	0.96	0.13
- H-AI	0.04	0.60	0.66	0.93	0.28
**Axillary brachial plexus**					
Fascia over conjoint tendon					
- H-H	0.00	0.08	0.03	0.67	0.12
- H-AI	0.00	0.23	0.16	1.00	0.25
**Erector spinae plane block**					
Transverse process					
- H-H	0.00	0.05	0.01	0.67	0.09
- H-AI	0.00	0.01	0.00	0.17	0.03
**Rectus sheath block**					
Rectus sheath					
- H-H	0.00	0.09	0.05	0.68	0.10
- H-AI	0.00	0.10	0.04	0.97	0.17
Peritoneum					
- H-H	0.00	0.09	0.03	1.00	0.17
- H-AI	0.00	0.30	0.29	0.97	0.19

Table 3 shows the comparative qualitative assessment for each structure (*n*=15 expert assessments from previous study^8^). These data are not available for six structures: anterior and middle scalene muscles, fascia over the conjoint tendon, erector spinae muscle group, rectus abdominis, and sartorius muscles. Two data points were not recorded for the qualitative assessment of AI system accuracy in identifying the radial nerve (*n*=13).

**Table 3:** Subjective assessment of artificial intelligence system accuracy with associated potential to modify the risk of adverse events, block failure, and subjective overall score of system performance. FN, false negative; FP, false positive; TN, true negative; TP, true positive.

	Accuracy rate % (n/n)			Adverse event % (n/n)			Nerve trauma % (n/n) ∗local anaesthetic systemic toxicity ^†^pneumothorax ^‡^peritoneal violation			Block failure % (n/n)			Subjective score
	(TP+TN)/Total	FP/Total	FN/Total	Increase	No change	Decrease	Increase	No change	Decrease	Increase	No change	Decrease	Mean (min–max; sd)
**Interscalene block**													
C5 nerve root	86.7 (13/15)	6.67 (1/15)	6.7 (1/15)	13.3 (2/15)	0 (0/15)	86.7 (13/15)	13.3 (2/15)	13.3 (2/15)	73.33 (11/15)	6.67 (1/15)	20.00 (3/15)	80.00 (12/15)	7.267 (0–10; 2.719)
C6 nerve root	100 (15/15)	0 (0/15)	0 (0/15)	6.7 (1/15)	20.0 (3/15)	73.3 (11/15)							
Scalenus anterior													
Scalenus medius													
**Axillary brachial plexus**													
Axillary artery	100 (15/15)	0 (0/15)	0 (0/15)	0 (0/15)	0 (0/15)	100 (15/15)	6.7 (1/15) ∗6.7 (1/15)	40.0 (6/15) ∗6.7 (1/15)	53.33 (8/15) ∗86.67 (13/15)	13.33 (2/15)	20.00 (3/15)	66.67 (10/15)	7.0 (2–9; 2.066)
Median nerve	86.7 (13/15)	13.3 (2/15)	0 (0/15)	6.7 (1/15)	20.0 (3/15)	73.3 (11/15)							
Musculocutaneous nerve	80.0 (12/15)	0 (0/15)	20.0 (3/15)	6.7 (1/15)	26.7 (4/15)	66.7 (10/15)							
Radial nerve	84.6 (11/13)	15.4 (2/13)	0 (0/13)	7.7 (1/13)	7.7 (1/13)	84.6 (11/13)							
Ulnar nerve	93.3 (14/15)	6.7 (1/15)	0 (0/15)	6.7 (1/15)	6.7 (1/15)	86.7 (13/15)							
Fascia conjoint tendon													
**Erector spinae plane block**													
Erector spinae muscle group							^**‡**^6.7 (1/15)	^**‡**^20.0 (3/15)	^**‡**^73.33 (11/15)	13.33 (2/15)	13.33 (2/15)	73.33 (11/15)	6.533 (1–10; 2.7)
Transverse process	80.0 (12/15)	0 (0/15)	20.0 (3/15)	6.7 (1/15)	26.7 (4/15)	66.7 (10/15)							
Pleura	93.3 (14/15)	0 (0/15)	6.7 (1/15)	13.3 (2/15)	13.3 (2/15)	73.3 (11/15)							
**Rectus sheath block**													
Rectus abdominis							^**†**^0 (0/15)	^**†**^20.00 (3/15)	^**†**^80.00 (12/15)	0 (0/15)	6.67 (1/15)	93.33 (14/15)	7.467 (5–9; 1.4)
Rectus sheath	100 (15/15)	0 (0/15)	0 (0/15)	0 (0/15)	13.3 (2/15)	86.7 (13/15)							
Peritoneum	100 (15/15)	0 (0/15)	0 (0/15)	0 (0/15)	20.0 (3/15)	80.0 (12/15)							
**Adductor canal block**													
Femoral artery	100 (15/15)	0 (0/15)	0 (0/15)	0 (0/15)	0 (0/15)	100 (15/15)	6.7 (1/15) ∗0 (0/15)	53.3 (8/15) ∗0 (0/15)	40.00 (6/15) ∗100.00 (15/15)	0 (0/15)	6.67 (1/15)	93.33 (14/15)	7.600 (2–9; 1.8)
Saphenous nerve	93.3 (14/15)	0 (0/15)	6.7 (1/15)	6.7 (1/15)	13.3 (2/15)	80.0 (12/15)							
Sartorius													
**Sciatic nerve block**													
Sciatic nerve	100 (15/15)	0 (0/15)	0 (0/15)	0 (0/15)	13.3 (2/15)	86.7 (13/15)	0 (0/15)	13.3 (2/15)	86.67 (13/15)	6.67 (1/15)	6.67 (1/15)	86.67 (13/15)	7.667 (1–10; 2.413)

## Discussion

This is the most comprehensive objective and quantitative evaluation of both human expert variability and AI system performance for evaluating ultrasound images relevant to regional anaesthesia. It is also the only study to present such data alongside qualitative and clinically orientated companion data. Human–human expert structure identification typically displayed a superior mean score (Dice/Hausdorff metric) than human–AI, although human–AI variation was lower.

**Fig 2:**
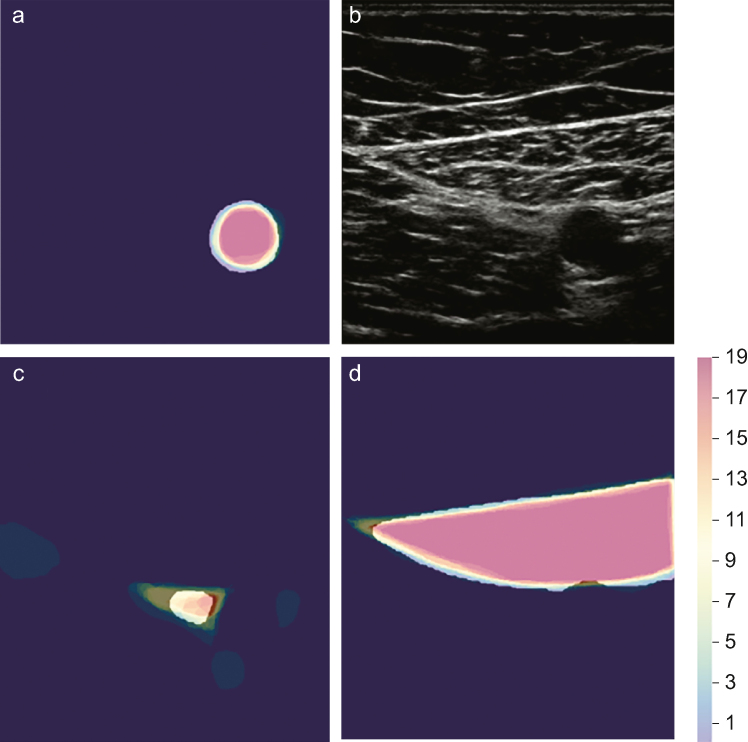
Visual depiction of the highest, intermediate, and lowest structure class annotations by the Dice metric. All annotations for (a) the femoral artery, (c) saphenous nerve, and (d) sartorius muscle were all taken from the same adductor canal block ultrasound scan (b). Colour scheme indicating number of human experts to include the pixel in their annotation (max=19). The white superimposed overlay shows ScanNav™.

**Fig 3:**
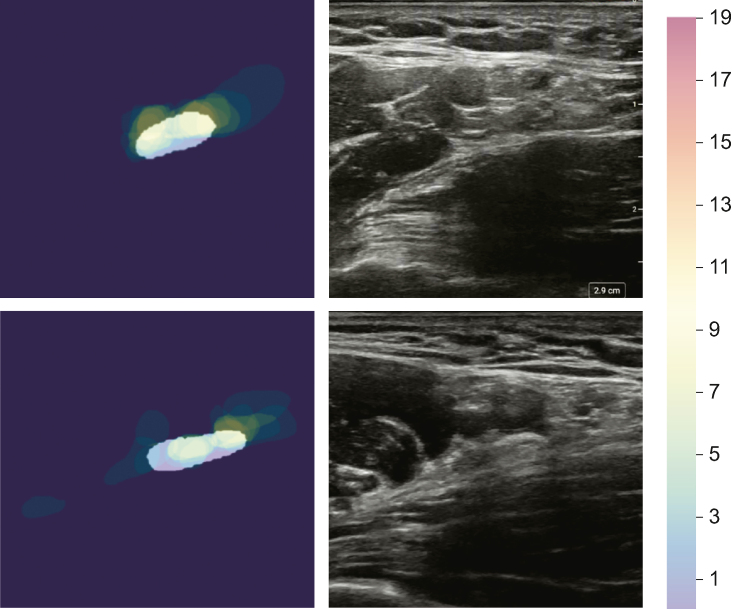
Examples of variability in annotation and associated artificial intelligence colour overlay for the radial nerve, the structure with the greatest variability. All annotations for the radial nerve in two images. The white superimposed overlay shows the ScanNav™ prediction.

Human experts displayed variability in the identification of anatomical structures defined as core (minimum) structures to be identified on the block view for the relevant peripheral nerve block. Expert agreement was highest with arteries and lowest with nerves, both of which are essential to efficacy and safety in UGRA. Visual interpretation of this variability by structure class is presented in Figure 2. Structures with lower level of agreement are often challenging to identify in clinical practice (e.g. radial nerve) and display anatomical variation (e.g. musculocutaneous nerve) (Fig. 3). Interpretation of ultrasound images is central to UGRA practice,^22^ but medical image interpretation is subjective, even amongst experts.^18^ Structural and functional dissimilitude in human anatomy has been described earlier in relation to UGRA.^23^^,^^24^ However, no prior studies have quantified such variability in UGRA expert sono-anatomical structure identification. The clinical significance of variability as demonstrated in this study is not yet clear, but future evaluations of AI technology should incorporate this factor rather than simply comparing AI performance to the pooled opinion of a small number of experts. All expert participants in this study regularly perform UGRA, and it is not possible to determine the extent to which this variability in structure identification influences efficacy or safety in practice. There is no clear threshold (for either Dice or Hausdorff metric) at which structure identification is deemed ‘satisfactory’, and any threshold might be different for different structure classes (e.g. nerve *vs* muscle). Nevertheless, based on these data, the assumption that expert opinion is uniform or definitive should be challenged.

ScanNav™, an AI system approved for clinical use in Europe and the USA, showed the same pattern of variability when compared with human experts. The highest level of agreement with human interpretation was seen for arteries and the lowest agreement for nerves. The mean score was typically lower, but with less variation. As with the differences in human–human agreement, the clinical significance of these differences is unclear.

Previous studies have attempted to quantify accuracy of AI systems for sono-anatomical structures relevant to regional anaesthesia.^7^^,^^8^^,^^11^^,^^16^^,^^17^^,^^25^, ^26^, ^27^ Many report the Dice metric (or similar), with results in a similar range, although typically the ground truth was determined by fewer (one to three) experts and only for a few selected structures. Three studies have used subjective evaluation of accuracy by UGRA experts,^7^^,^^8^^,^^11^ two of which are our prior evaluations of ScanNav™. Given the human expert variability demonstrated here, it is not clear whether comparison with a small number of experts is appropriate or whether quantitative assessment (utilising the Dice metric or similar) is useful. The authors hypothesise that non-experts display lower mean scores and greater variability than experts, although many still use these techniques in their clinical practice (often independently). It is therefore unclear what level of performance is required for AI systems (or humans) to be suitable for clinical use. As shown in this study, systems have higher levels of agreement for some structures (e.g. sartorius muscle mean Dice metric 0.92) than for others (e.g. radial nerve mean Dice metric 0.27). This raises the question of whether systems should be approved in their entirety, or on a structure-by-structure basis. In either case, data for a full set of structures should be included in an assessment of a system in its entirety rather than simply a subset of the data.

The subjective and qualitative data show similarities in the patterns of system accuracy (e.g. accuracy is often lowest for nerves). However, there is less granularity in these qualitative data (e.g. lower rate of structure identification is less pronounced for the radial nerve compared with the quantitative data). Given the variability in objective data from the human experts, it is not certain whether this level of granularity is useful information. Despite limitations to the AI structure identification, there were few cases of perceived increased risk for adverse events or block failure. Because of the emerging nature of this field and uncertainty over what level and type of data are required, the authors suggest that an optimal approach is to present these data in a standardised format as in this study. This will allow consistent evaluation of different systems and comparison of one system with another.

Ultrasonography was introduced and used in clinical practice before data confirmed its efficacy or safety. Several benefits have since been proven, although it is still not established that use of ultrasound reduces the incidence of nerve injury in UGRA.^28^ Similarly, AI is a rapidly evolving field with an emerging influence on clinical practice. The clinical community must familiarise themselves with the AI field and its common methodologies (e.g. Dice/Hausdorff metrics) to evaluate the level of evidence required when implementing AI systems in anaesthetic practice.

There is currently no uniform system of evaluation or agreement on what approach is sufficiently robust to evaluate emerging AI devices in UGRA prior to approval for clinical use. Technical (quantitative) methods of system evaluation lack clinical context, whereas clinical evaluations can be subjective and lack the same level of detail. These data demonstrate a consistent framework to evaluate novel AI devices in UGRA, combining objective/quantitative with subjective/clinical assessments.

An open-access repository of anonymised ultrasound videos and images, with an independent ‘ground truth’ evaluation using an agreed set of recommended structures,^4^^,^^5^ could be used in future to evaluate the performance of AI devices (or assess the performance of other human operators). Such an approach is used in other fields of AI applied to image interpretation.^29^, ^30^, ^31^ Expert assessment of scans, such as that presented here, can provide a benchmark against which performance of non-experts can be compared. Consistent reporting, in accordance with existing and forthcoming guidelines (e.g. DECIDE-AI and STARD-AI),^21^^,^^32^ could further enable transparent and meaningful comparison between studies and better inform clinicians. This strategy of evaluation would allow standardisation, a concept that has become popular in other aspects of UGRA.^4^^,^^5^^,^^33^^,^^34^

Finally, objective measures of evaluation do not necessarily translate to clinical utility or patient benefit. Therefore, validation of devices would benefit from both objective and subjective measures, from a clinical and technical perspective, before proceeding to real-world clinical trials. These issues must be addressed urgently as AI systems are already available in clinical practice. The initial systems focus on supporting ultrasound scan acquisition and interpretation by non-experts, deskilling a key element of UGRA. This is a positive aspiration for delivery of UGRA by junior anaesthetists or by other specialities (e.g. emergency medicine). However, although AI systems can provide guidance, underlying knowledge is still required of the operator. It is therefore paramount they are deployed in this way, as the clinician utilising the system is likely to be less experienced. The anaesthetic workforce must be engaged and well-informed if they are to guide development and adoption of this technology safely and effectively.

The authors recognise limitations to this study. Firstly, the experts analysing the images did not acquire the scans. A major component of information-gathering in ultrasound image interpretation is the dynamic scanning process, which allows the operator to track structures in relation to one another, determine features such as anisotropy, and gain tactile feedback. This might have been particularly important for nerves in the axillary brachial plexus region given the lower scores. Real-time performance of scanning was not available to the expert participants in this study, although they were able to review the video repeatedly and without a time limit. However, had each participant been able to acquire and interpret their own scans, even on the same subject, they would almost certainly result in a different image and thus the study would lack consistency. Thus, these analyses were based on individual still frame images as opposed to real-time ultrasound, whereby structure identification can become clearer in a preceding or subsequent frame. Despite drawbacks, if the same (pre-recorded) data from expert analysis is to be used for different AI systems, this allows a consistent evaluation for each one.

A second limitation is that expert participants commented that some images were difficult to interpret, reporting that they would typically acquire superior images for their own UGRA practice. The scans underwent a quality control process as part of the acquisition protocol in the previous study; experts (not involved in this study) acquired the scans and a panel of three further experts assessed whether the scan was adequate for clinical use. The discrepancy in opinion might reflect variation in what an expert considers to be an acceptable scan. In addition, it is important that AI systems accurately interpret suboptimal scans, which could be where their value is greatest. Thus, evaluation of model performance on suboptimal scans, and scans of subjects with challenging or varied anatomy, should necessarily form part of validation.

A third limitation is that data entries where a structure was not annotated were included in the analysis (interpreted as ‘not present’). Although participants were instructed to omit annotation for structures they felt were not present, they did not have the opportunity to explicitly state this. Thus, individual structure annotations could have been missed in error rather than because the expert believed it to be ‘not present’. However, the scans had undergone a quality control process in the previous study, whereby a panel of three experts determined that the view was appropriate and excluded any atypical anatomy. Although AI systems might not be as accurate as human experts in identifying specific structures, this element of ‘human error’ (whereby a structure annotation is inadvertently missed) does not occur in machines, making them more accurate and consistent in that regard. Furthermore, it is notable that most structures had a Dice score range that started at 0, meaning that there was no overlap between at least two of the expert annotations. This includes structures that one might expect to identify clearly (e.g. the axillary artery). In such cases, ‘human error’ again might have contributed to identifying/annotating the wrong structure (e.g. axillary vein rather than artery, or scalenus posterior *vs* anterior if the scan orientation is misinterpreted despite it being labelled).

Limitations to qualitative and subjective data have been discussed,^8^ although the authors recognise that fewer (and different) experts provided the qualitative data. Future studies should aim to concurrently obtain qualitative/subjective and quantitative/objective data from the same experts.

Finally, it is not possible to determine whether the differences in these data translate to meaningful differences in the clinical setting; further work to investigate the clinical implications is necessary.

### Conclusions

We performed a quantitative and objective evaluation of the variability of human experts and an AI system when identifying structures on ultrasound images relevant to UGRA, presenting this alongside a qualitative and subjective evaluation of the same ultrasound and AI data. Both humans and the AI system showed the greatest level of consistency when identifying arteries, followed by muscles, and then nerves. Human–human mean scores tended to be higher, whereas human–AI scores tended to show less variation. The clinical significance of these differences is yet to be determined. AI is a rapidly emerging field; greater understanding and clinician engagement is required to inform the development, evaluation, and adoption of these novel devices. The evaluation structure described here, despite the acknowledged limitations, could form a framework to evaluate such novel AI devices in UGRA to allow meaningful comparison between them.

## Authors’ contributions

Study concept and design: JSB, RM

Participant recruitment and data collection: JSB, OL, JL, DBSL, BB, AJRM, AP, AT, HH

Manuscript preparation and editing: all authors

## Declarations of interest

JSB is a Senior Clinical Advisor for Intelligent Ultrasound Limited (IUL), UK, receiving research funding and honoraria. RM is employed by IUL. DBSL is a Clinical Advisor for IUL, receiving honoraria. BB declares honoraria from IUL. AJRM declares honoraria from IUL and GE Healthcare, and is the president of Regional Anaesthesia UK. AP declares honoraria from GE Healthcare, USA, and Pacira, USA, and is the immediate past-president of Regional Anaesthesia UK. AT declares honoraria from IUL. JAN is a senior scientific advisor for IUL. The device studied (*ScanNav*^*TM*^ *Anatomy Peripheral Nerve Block*) is a product of IUL.

## Funding

Maurice Freeman Barema & Association of Anaesthetists grant (HMR03690/HM00.01) to JSB; 10.13039/501100000769University of Oxford stipend (Intelligent Ultrasound Limited) to JSB.
