# Supplementary material for: Variability between human experts and artificial intelligence in identification of anatomical structures by ultrasound in regional anaesthesia: a framework for evaluation of assistive artificial intelligence
Source: Br J Anaesth. 2023 Oct 27;132(5):1063–72. doi: 10.1016/j.bja.2023.09.023 (PMC11103080; doi:10.1016/j.bja.2023.09.023)
Supplement: Multimedia component 1 [file mmc1.pdf]

Variability between human experts and artificial intelligence for identification of anatomical structures on ultrasound in regional anaesthesia: a framework evaluation of assistive artificial intelligence

Supplementary material

Authors

James S Bowness[1,2], Robert Morse[3], Owen Lewis[2], James Lloyd[2], David Burckett-St Laurent[4], Boyne Bellew[5,6], Alan JR Macfarlane[7,8], Amit Pawa[9,10], Alasdair Taylor[11], J Alison Noble[12], Helen Higham[1,13]

Author Institutions

1. Oxford Simulation, Teaching and Research Centre, University of Oxford, Oxford, UK
2. Department of Anaesthesia, Aneurin Bevan University Health Board, Newport, UK
3. Intelligent Ultrasound, Cardiff, UK
4. Department of Anaesthesia, Royal Cornwall Hospitals NHS Trust, Truro, UK
5. Department of Surgery & Cancer, Imperial College London, London, UK
6. Department of Anaesthesia, Imperial College Healthcare NHS Trust, London, UK
7. Department of Anaesthesia, NHS Greater Glasgow & Clyde, Glasgow, UK
8. School of Medicine, Dentistry & Nursing, University of Glasgow, Glasgow, UK
9. Department of Anaesthesia, Guy's & St Thomas' NHS Foundation Trust, London, UK
10. Faculty of Life Sciences and Medicine, King's College London, London, UK
11. Department of Anaesthesia, NHS Tayside, Dundee, UK
12. Institute for Biomedical Engineering, University of Oxford, Oxford, UK
13. Department of Anaesthesia, Oxford University Hospitals NHS Foundation Trust, Oxford, UK

Anatomical Structures Identified

PNB peripheral nerve block; **ISB** interscalene level brachial plexus block; **AxBP** axillary level brachial plexus block; **ESP** erector spinae plane block; **RSB** rectus sheath block; **ACB** adductor canal block; **SNB** popliteal level sciatic nerve block (*****=enclosed structure annotation, ⁺=line annotation)

PNB	*Nerve	*Artery	⁺Fascial/ Serosal Plane	⁺Bone	*Muscle
**ISB**	C5 nerve root<br>C6 nerve root				Scalenus anterior<br>Scalenus medius
**AxBP**	Musculocutaneous nerve<br>Median nerve<br>Ulnar nerve<br>Radial nerve	Axillary artery	Fascia over conjoint tendon		
**ESP**				Transverse process	Erector spinae muscle group
**RSB**			Rectus sheath (anterior layer)<br>Rectus sheath (posterior layer)<br>Peritoneum		Rectus abdominis
**ACB**	Saphenous nerve	Femoral artery			Sartorius
**SNB**	Sciatic nerve				

Scan Subject Demographic Data

Model ID	Block	Age	Sex	BMI
M01	AX	42	F	27.3
M03	POP	56	M	31.6
M04	RS	42	M	24.3
M04	ADD	42	M	24.3
M06	AX	35	F	36
M07	ESP	23	F	38.4
M07	POP	23	F	38.4
M12	AX	31	F	28.7
M12	ADD	31	F	28.7
M12	ESP	31	F	28.7
M13	POP	58	F	25.7
M14	ESP	36	F	32.3
M17	ADD	61	M	28.6
M22	ESP	53	M	30.7
M27	IS	58	F	30.1
M27	RS	58	F	30.1
M29	ADD	47	M	21.6
M30	IS	63	M	24.4
M31	ESP	64	F	21.7
M33	AX	34	M	37.1
M33	IS	34	M	37.1
M33	POP	34	M	37.1
M34	IS	27	M	22.8
M34	POP	27	M	22.8
M36	RS	27	M	34.6
M38	IS	28	M	27.4
M38	RS	28	M	27.4
M39	RS	25	F	19.7
M40	AX	25	F	23.4
M40	ADD	25	F	23.4

Instructions for Annotation

In the following ultrasound images, you will be asked to annotate structures using enclosures and lines.

Enclosures

- Generally, when using an enclosure, you will draw a closed shape around the perimeter of an object. You should aim to join your line
- The perimeter of some structures may include one or more edges of the ultrasound image. In these cases, you do not need to 'draw' a line along the edge – this will be done automatically.
- Any annotation outside the ultrasound image will be 'clipped'. Therefore, if you believe the structure being annotated extends to the edge of the image, you can extend your annotation outside the image to ensure you have reached the edge
- Artery enclosures should be the perimeter of the lumen
- Nerve enclosures should be the outer perimeter

Lines

- The line should be drawn over the most prominent (brightest) part of the anatomical structure being annotated (e.g., pleura/peritoneum, fascial layer or bone)
- You should only annotate parts of the structure that can be seen (e.g., if a layer of the rectus sheath is visible on one side of the screen but fades out and the other end can't be seen with certainty, stop you annotation when the structure can no longer be confidently identified)
- Any annotation outside the ultrasound image will be 'clipped'. Therefore, if you believe the structure being annotated extends to the edge of the image, you can extend your annotation outside the image to ensure you have reached the edge.

Please Note

- The cursor is displaced from the pen tip by a few pixels, so use the cursor to draw (hovering over the screen shows you where the cursor is before you draw)
- The structures you will be asked to annotate for each block, with any specific additional information required, are listed below

Interscalene

Enclosures

- Anterior scalene (annotate the external boundary of the muscle; same for all muscles)
- Middle scalene
- C5 nerve root (annotate the external boundary of the nerve; same for all nerves)
- C6 nerve root

Axillary

Enclosures

- Axillary artery (annotate the lumen of the vessel (not wall); same for all blood vessels)
- Axillary vein
- Median nerve
- Musculocutaneous nerve
- Radial nerve
- Ulnar nerve

Lines

- Conjoint tendon (annotate the fascia overlying the conjoint tendon)

Erector Spinae

Enclosures

- Erector spinae muscle group; annotate the superficial and deep borders of the erector spinae muscle group (the perimeter of this structures may include an edge/s of the ultrasound image - you do not need to continue your line down the edge of the image)

Lines

- Transverse process (annotate the posterior/superficial surface of the transverse process)

Rectus Sheath

Enclosures

- Rectus abdominis (the perimeter of this structure may include an edge/s of the ultrasound image - you do not need to continue your line down the edge of the image)

Lines

- Rectus sheath (anterior layer; annotate the anterior layer of the sheath)
- Rectus sheath (posterior layer; annotate the posterior layer of the sheath)
- Peritoneum (annotate the peritoneum)

Adductor Canal

Enclosures

- Femoral artery
- Sartorius (the perimeter of this structure may include an edge/s of the ultrasound image - you do not need to continue your line down the edge of an image)

- Saphenous nerve/nerve complex

Popliteal
Enclosure
- Sciatic nerve (as the single structure or the point at which the nerve is dividing, as one overall outline)
